# The host response to the probiotic *Escherichia coli *strain Nissle 1917: Specific up-regulation of the proinflammatory chemokine MCP-1

**DOI:** 10.1186/1471-2350-6-43

**Published:** 2005-12-13

**Authors:** Sya N Ukena, Astrid M Westendorf, Wiebke Hansen, Manfred Rohde, Robert Geffers, Sina Coldewey, Sebastian Suerbaum, Jan Buer, Florian Gunzer

**Affiliations:** 1 German Research Centre for Biotechnology, Mucosal Immunity Group, Mascheroder Weg 1, 38124 Braunschweig, Germany; 2 German Research Centre for Biotechnology, Department of Microbiology, Mascheroder Weg 1, 38124 Braunschweig, Germany; 3 Medical School Hannover, Department of Medical Microbiology and Hospital Epidemiology, Carl-Neuberg-Str.1, 30625 Hannover, Germany

## Abstract

**Background:**

The use of live microorganisms to influence positively the course of intestinal disorders such as infectious diarrhea or chronic inflammatory conditions has recently gained increasing interest as a therapeutic alternative. *In vitro *and *in vivo *investigations have demonstrated that probiotic-host eukaryotic cell interactions evoke a large number of responses potentially responsible for the effects of probiotics. The aim of this study was to improve our understanding of the *E. coli *Nissle 1917-host interaction by analyzing the gene expression pattern initiated by this probiotic in human intestinal epithelial cells.

**Methods:**

Gene expression profiles of Caco-2 cells treated with *E. coli *Nissle 1917 were analyzed with microarrays. A second human intestinal cell line and also pieces of small intestine from BALB/c mice were used to confirm regulatory data of selected genes by real-time RT-PCR and cytometric bead array (CBA) to detect secretion of corresponding proteins.

**Results:**

Whole genome expression analysis revealed 126 genes specifically regulated after treatment of confluent Caco-2 cells with *E. coli *Nissle 1917. Among others, expression of genes encoding the proinflammatory molecules monocyte chemoattractant protein-1 ligand 2 (MCP-1), macrophage inflammatory protein-2 alpha (MIP-2α) and macrophage inflammatory protein-2 beta (MIP-2β) was increased up to 10 fold. Caco-2 cells cocultured with *E. coli *Nissle 1917 also secreted high amounts of MCP-1 protein. Elevated levels of MCP-1 and MIP-2α mRNA could be confirmed with Lovo cells. MCP-1 gene expression was also up-regulated in mouse intestinal tissue.

**Conclusion:**

Thus, probiotic *E. coli *Nissle 1917 specifically upregulates expression of proinflammatory genes and proteins in human and mouse intestinal epithelial cells.

## Background

Probiotic microorganisms have traditionally been characterized as viable nutritional agents conferring benefits to the health of the human host [1]. The definition of the term "probiotic" has evolved through the years. The latest and most appropriate definition describes probiotics as "live microorganisms which when administered in adequate amounts confer a health benefit on the host" [2]. Beneficial activities of probiotics most likely result from complex interactions of the microorganisms with the intestinal microflora and the gut epithelium of the individual [3]. A proposed mechanism by which probiotics mediate their effects is the modulation of the innate immune response both to antiinflammatory [4] and proinflammatory directions. Furthermore, probiotic bacteria have been shown to enhance the adaptive immune response and antibody formation [7,8]. Inhibition of adherence of attaching and effacing organisms [9], modulation of the mucosal barrier function [10,11] as well as inhibition of neutrophil migration [12] may also be important mechanisms whereby probiotics can impact in intestinal diseases.

Next to lactic acid bacteria and the probiotic yeast *S. boulardii*, the non-pathogenic *E. coli *strain Nissle 1917 (EcN) of serotype O6:K5:H1 is one of the best characterized probiotics. EcN was originally isolated by the army surgeon Dr. Alfred Nissle in 1917 from the feces of a soldier who did not develop diarrhea during a severe outbreak of shigellosis [13]. A controlled clinical trial published recently implies probiotic EcN being as effective as standard medication with a dose of 1,5 g/day mesalazine in remission maintenance of ulcerative colitis. In this study recurrance rates were 33.9% for mesalazine treatment compared to 36.4% for treatment with EcN (Mutaflor^®^) [14]. Recently, we have demonstrated, that recombinant EcN had no effect on migration, clonal expansion and activation status of specific CD4^+ ^T cells, neither in healthy mice nor in animals with acute colitis [15]. Despite the successful therapeutic applications of EcN, only limited information is available about the beneficial traits contributing to the strains' probiotic character. Several strain specific characteristics have been detected so far including expression of two microcins [16], presence of six iron-uptake systems or lack of defined virulence factors [17]. Moreover, EcN exhibits a unique semirough lipopolysaccharide phenotype, responsible for its serum sensitivity [18,19]. All these properties might be advantageous for EcN in competing with other colonic bacteria or adapting to the intestinal situation. However, the mechanisms underlying the probiotic nature, especially at the molecular level, yet have to be elucidated.

In this study we aimed to analyze the genomic expression program initiated by the interaction of probiotic EcN with human intestinal epithelial cells. Our results demonstrate a transient proinflammatory signaling of human and mouse intestinal epithelial cells illustrated by induced gene expression of MCP-1, MIP-2α and MIP-2β after treatment with EcN.

## Methods

### Cell culture conditions

The human colon adenocarcinoma cell lines Caco-2 [20] and Lovo [21] were maintained in IMDM cell culture medium (Invitrogen, Karlsruhe, Germany) containing 10% fetal calf serum (PAA Laboratories, Cölbe, Germany) and 250 μg/ml penicillin/streptomycin (Invitrogen) at 37°C in a cell culture incubator. Caco-2 cells were used from passage 12 – 26. Cells were split twice a week at a ratio of 1:3. 4 – 8 × 10^5 ^cells per well were seeded in six well plates (Nunc, Wiesbaden, Germany) and cultured for approximately four days until confluence.

### Animals

BALB/c mice were obtained from Harlan (Borchen, Germany). The animal experiments reported here were permitted from the district authority of Braunschweig and were conducted according to the German animal protection law.

### Preparation of bacteria

*E. coli *strain Nissle 1917 was isolated from a tablet of Mutaflor^® ^(Ardeypharm, Herdecke, Germany) cultured on MacConkey plates (Oxoid, Wesel, Germany). The isolate was serotyped and confirmed by EcN specific PCR [22]. *E. coli *K12 laboratory strain MG1655 was kindly provided by Ulrich Dobrindt (Institute for Molecular Infection Biology, Würzburg, Germany). Both strains were grown overnight in Luria Bertani (LB) medium (Invitrogen) at 37°C on a shaker. The cultures were then diluted 1:100 in 50 ml prewarmed IMDM medium containing 10% fetal calf serum and grown at 37°C. Bacteria were harvested in late logarithmic phase, after reaching an OD_600 _= 1.

### Coculture of cell lines

Confluent Caco-2 and Lovo cells (0.4 – 1 × 10^7 ^cells per well) were washed with phosphate buffered saline (PBS) and cocultured with a low bacterial MOI (multiplicity of infection) of 1 (1.3 – 3.3 × 10^6 ^CFU/ml) in IMDM medium containing 10% fetal calf serum at 37°C in a cell culture incubator for 6 hours. For time-dependent expression experiments conditioned media was collected by centrifugation after coincubation for 6 hours, redissolved in 3 ml IMDM medium with 10% fetal calf serum containing 200 μg/ml gentamicin (Sigma-Aldrich, Taufkirchen, Germany) and 250 μg/ml penicillin/streptomycin (Invitrogen), and used to incubate the Caco-2 cells for another 42 hours. Prior to RNA isolation cells were washed twice with PBS.

### Tissue coculture

Experimental setup was performed following a modified protocol described by Cima et al. [23]. In brief, small intestine was dissected, washed and opened logitudinally, cut into 5 mm long pieces and washed with PBS/DTT for 15 min shaking at 37°C to remove the excess mucus. Tissue pieces were then washed with PBS followed by two washing steps with HBSS/2% fetal calf serum. 3 – 5 tissue pieces per well were placed in a 24 well plate in IMDM containing 10% fetal calf serum without antibiotics. Bacteria were applied at 5 × 10^5 ^CFU/ml.

### Cocultivation of Caco-2 cells with inactivated bacterial pellets or bacteria conditioned media

Bacteria were grown overnight as described above. Overnight culture was diluted in IMDM with 10% fetal calf serum. For coincubation experiments with conditioned media (CM), bacterial suspensions were again incubated for 5 hours to an OD_600 _= 1 at 37°C on a shaker and diluted to 1 × 10^8 ^CFU/ml in IMDM medium containing 10% fetal calf serum. CM was centrifuged and supernatants were sterile filtered. Caco-2 cells were incubated with inactivated bacteria or CM for 6 hours. For experiments with inactivated bacteria, an aliquot of the bacterial suspension was taken at an OD_600 _= 0.5 and centrifuged. The bacterial pellet was fixed in 4% paraformaldehyde for one hour at room temperature, washed four times with PBS, redissolved in IMDM medium with 10% fetal calf serum and adjusted to an MOI of 2. Aliquots of the fixed bacteria were plated onto Müller-Hinton-agar to test for sterility.

### RT-PCR

RNA from cells and tissue was extracted using TriFast FL (PEQLAB, Erlangen, Germany) according to the manufacturer's protocol, following DNAse I digestion with DNA-free^® ^(Ambion, Huntingdon Cambridgeshire, UK). Samples were pooled prior to reverse transcription with 200 U Superscript II^® ^(Invitrogen) for 45 minutes at 42°C in 20 μl assays containing 1 μl Oligo dT-primers and 10 mM deoxynucleoside triphosphates (dNTPs). Conventional PCR was performed with AmpliTaq Gold^® ^DNA polymerase (Applied Biosystems, Darmstadt, Germany) in 20 μl assays containing 0.25 pmol primers and 0.5 mM dNTP using the following cycling conditions: Activation of Taq polymerase for 10 minutes at 95°C, followed by a first round of 10 cycles each consisting of a 30 second denaturing interval at 95°C, a 90 second annealing step at the respective primer T_m _and a primer extension at 72°C for again 90 seconds. This first amplification step was followed by a second round of 27 cycles, consisting of 15 seconds denaturation, 45 seconds annealing and 90 seconds extension. PCR products were separated in 2% agarose gels and visualized by ethidium-bromide staining. All primers used in this study are listed in table 1.

### DNA microarray hybridization

Quality and integrity of the total RNA isolated from 1 × 10^6 ^cells was controlled by running all samples on an Agilent 2100 Bioanalyzer (Agilent Technologies, Waldbronn, Germany). For biotinylated target synthesis, RNA was labeled using standard protocols supplied by the manufacturer (Affymetrix, Santa Clara, CA). Briefly, 5 μg total RNA was converted to dsDNA using 100 pmol of a T7T23V primer (Eurogentec, Seraing, Belgium) containing a T7 promoter. The cDNA was then used directly in an *in vitro *transcription reaction in the presence of biotinylated nucleotides. The concentration of biotin-labeled cRNA was determined by UV absorbance. In all cases, 12.5 μg of each biotinylated cRNA preparation were fragmented and placed in a hybridization cocktail containing four biotinylated hybridization controls (BioB, BioC, BioD, and Cre) as recommended by the manufacturer. Samples were hybridized to an identical lot of Affymetrix HG_U133A arrays for 16 hours. After hybridization, the GeneChips were washed, stained with strepavidin-phycoerythrin and read using an Affymetrix GeneChip fluidic station and scanner.

### Data analysis

Analysis of microarray data was performed using Affymetrix Microarray Suite 5.0, Affymetrix MicroDB 3.0 and the Affymetrix Data Mining Tool 3.0. For normalization all array experiments were scaled to a target intensity of 150, otherwise using the default values of the Microarray Suite. Results were filtered as follows: Genes are considered strongly regulated when their fold change is greater than or equal 2 or less than or equal -2, the statistical parameter for a significant change is less than 0.01 (change p-value for changes called increased) or greater than 0.99 (change p-value for changes called decreased). Additionally, the signal difference of a certain gene should be greater than 100. Genes are considered as weakly regulated when their fold change is greater than or equal 1.5 or less than or equal -1.5, the statistical parameter for a significant change is less than 0.001 or greater than 0.999 and the signal difference of a certain gene should be greater than 40.

### Real-time RT-PCR

Total RNA preparation and cDNA synthesis were performed as described for RT-PCR. Quantitative real-time RT-PCR was done with an ABI PRISM cycler (Applied Biosystems, Foster City, CA) using SYBR Green PCR kit from Stratagene and specific primers optimized to amplify 90 – 250 bp fragments from the genes under investigation (Tab. 1). A threshold was set in the linear part of the amplification curve, and the number of cycles needed to reach it was calculated for every gene. Relative mRNA levels were determined by using included standard curves for each individual gene and further normalization to the housekeeping gene RPS9. Melting curves established the purity of the amplified band.

### Cytokine analysis by CBA

Quantification of cytokines was performed using the BD^® ^Cytometric Bead Array Human Chemokine Kit I with antibodies specific for IL-8, RANTES, MIG, MCP-1 and IP-10 (BD Biosciences, Heidelberg, Germany). Supernatants of 3–5 tissue culture wells were pooled and data were acquired as described previously [24] by flow cytometry with a FACSCalibur^® ^flow cytometer (BD Biosciences) using 2-color detection. Data analysis was performed by BD Bioscience Cytometric Bead Array software.

### Field emission scanning electron microscopy

Caco-2 cells were seeded on 22 mm Biocoat Collagen I coated coverslips (BD Biosciences) in six well plates and grown until confluence as described above. After coincubation, cells were washed three times with 1 × PBS and fixed with a fixation solution containing 5% formaldehyde and 2% glutaraldehyde in cacodylate buffer (0.1 M cacodylate, 0.01 M CaCl_2_, 0.01 M MgCl_2_, 0.09 M sucrose, pH 6.9), washed with cacodylate buffer and then with TE-buffer (10 mM TRIS, 2 mM EDTA, pH 6.9). Finally, cells were scratched from the wells, pelleted and embedded in 2% aqueous agar before dehydrating with a graded series of acetone (10, 30, 50, 70, 90, 100%) on ice for 30 min at each step. Samples in 100% acetone were allowed to reach room temperature before another change of 100% acetone. They were then subjected to critical-point drying with liquid CO_2 _(CPD 030, BAL-TEC, Schalksmühle, Germany). The dried cells were covered with a gold film of about 10 nm by sputter coating (SCD040, BAL-TEC) before examination in a field emission scanning electron microscope Zeiss DSM 982 Gemini (Carl Zeiss, Oberkochen, Germany) using the Everhart Thornley SE detector and the inlens detector in a 50:50 ratio at an acceleration voltage of 5 kV. Data were stored digitally on MO-disks.

## Results

### Genome-wide expression analysis of Caco-2 cells treated with *E. coli *Nissle 1917

**Figure 1:**
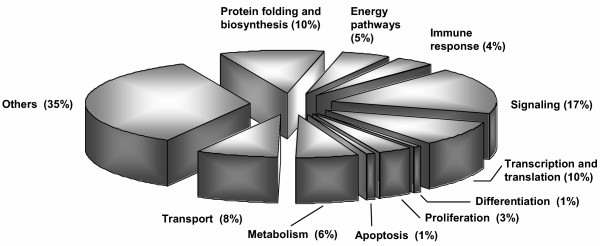
**Classification of genes regulated in confluent Caco-2 cells by coculture with EcN for 6 hours. **Analysis of microarray data from two independent experiments resulted in 126 genes being regulated. These genes could be assigned to different classes according to their involvement in biological processes (percent by number of genes per class).

Among the 22,000 genes and expressed sequence tags present on Affymetrix HG_U133A arrays, we identified 138 sequences differentially expressed upon treatment of confluent Caco-2 cells with EcN. Due to some genes being repeatedly present on the array, we finally found a total of 126 individual genes to be regulated. They belonged to a wide variety of gene classes (Fig. 1). From the coding sequences that could be assigned to one of these classes, 17% pertained to signaling pathways. The remainder of the genes encoded for molecules involved in protein folding and biosynthesis (10%), transcription and translation (10%), transport (8%), metabolism (6%) as well as energy pathways (5%), immune response (4%) proliferation (3%), differentiation (1%) and apoptosis (1%). A better part of them, like genes coding for hypothetical proteins and unannotated ORFs, could not be allocated to one of the classes and was therefore assigned as others (35%). Selected genes of each class, that showed at least a two fold regulation in one of the two experiments, were summarized in table 2. 9 of these genes were selected for further investigations. They were choosen on the basis of both, prominent changes in their gene expression profiles and their putative biological implication at the outset of our study. Significantly elevated mRNA levels were found for dual-specificity phosphatase 5 (DUSP5), chemoattractant protein-1 ligand 2 (MCP-1), macrophage inflammatory protein-2 alpha (MIP-2α), macrophage inflammatory protein-2 beta (MIP-2β), tumor necrosis factor alpha induced protein 3 (TNFαIP3) and ets domain transcription factor ELF3. Vascular endothelial growth factor (VEGF), active in angiogenesis, vasculogenesis and endothelial cell growth, was up-regulated as well. Yet another gene, up-regulated by EcN treatment, was human nuclear factor of kappa light chain gene enhancer in B cells inhibitor alpha (NFκBIA), which inhibits the action of NF-κB [25]. Downregulation of gene expression was observed for epithelial membrane protein 3 (EMP3). The mRNA expression levels of these genes were validated by real-time RT-PCR. The entire data set of our microarray experiments is accessible as MIAME format online with accession number GSE2232 [48].

### Real-time RT-PCR to confirm data obtained with gene expression analysis arrays

**Figure 2:**
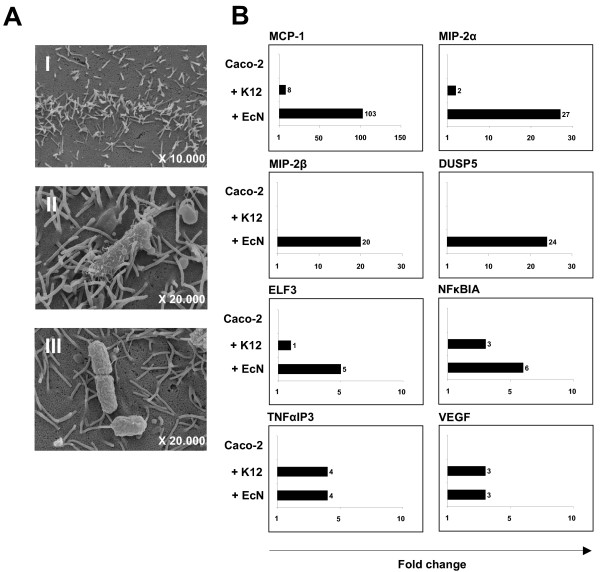
**Electron microscopy of coculture experiments and real-time RT-PCR analysis. **(A) Electron microscopy of confluent Caco-2 cells (I) cocultured with *E. coli *MG1655 (II) or EcN (III) was performed in order to visualize the interaction between cells and bacteria. (B) Validation of mRNA expression levels of Caco-2 cells by quantitative real-time RT-PCR. After 6 hours of coincubation with either *E. coli *MG1655 (+ K12) or EcN (+ EcN), total RNA was isolated, reversely transcribed, and relative mRNA expression levels for selected genes and the housekeeping gene RPS-9 (as internal control) were analyzed in duplicate real-time RT-PCR assays. Relative mRNA amounts were normalized with respect to expression levels of untreated Caco-2 cells (fold change = 1). The figure is representative of two independent experiments. MCP-1 indicates chemoattractant protein-1 ligand 2; MIP-2α, macrophage inflammatory protein-2 alpha; MIP-2β, macrophage inflammatory protein-2 beta; DUSP5, dual specificity phosphatase 5; NFκBIA, nuclear factor of kappa light polypeptide gene enhancer in B cell inhibitor, alpha; TNFαIP3, tumor necrosis factor, alpha-induced protein 3; VEGF, vascular growth factor and ELF3, E74-like factor 3 (ets domain transcription factor, epithelial-specific).

Electron micrographs of the coculture experiments are shown in figure 2A. Panel A I illustrates untreated Caco-2 cells. Cells coincubated with *E. coli *MG1655, serving as control strain, and EcN are depicted in panels A II and A III, respectively. Array data for MCP-1, MIP-2α, MIP-2β, DUSP5, ELF3 and NFκBIA were verified by real-time RT-PCR (Fig. 2B). The strongest up-regulation of mRNA expression, compared to control cells, could be detected for MCP-1. There was a 103 fold increase, compared to an mRNA level elevated only 8 fold after treatment with *E. coli *MG1655. Additionally, gene expression levels of MIP-2α, MIP-2β and DUSP5 were more than 20 fold higher than the controls, when Caco-2 cells were treated with EcN. Array data obtained for TNFαIP3 and VEGF could also be confirmed by real-time RT-PCR, but the increase in mRNA levels was almost identical for Caco-2 cells treated with *E. coli *MG1655 or EcN. Therefore, up-regulation of TNFαIP3 and VEGF could not be considered to be an EcN specific effect. We were not able to verify down-regulation of EMP3 by real-time RT-PCR as indicated by our array data (data not shown). In summary, the regulation pattern of EcN-treated Caco-2 cells compared to treatment with *E. coli *MG1655 after 6 hours coculture suggested a specific EcN-mediated up-regulation of MCP-1, MIP-2α, MIP-2β, DUSP5, NFκBIA and ELF3 expression in human intestinal epithelial cells.

### Detection of MCP-1 in Caco-2 coculture supernatants by CBA analysis

**Figure 3:**
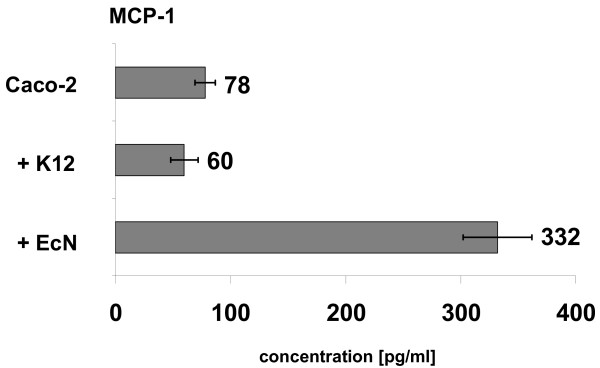
**MCP-1 secretion of Caco-2 cells after treatment with EcN. **Caco-2 cells were cocultured with *E. coli *MG1655 (+ K12) and EcN (+ EcN) for 6 hours. Culture supernatants were analyzed for several cytokines using the cytometric bead array kit I from BD Bioscience. Cytokine quantity is depicted as pg/ml. Data are presented as mean from two independent experiments.

Supernatants from Caco-2 cells cocultured with EcN and *E. coli *MG1655 were analyzed for several cytokines. Compared to untreated Caco-2 cells and cells treated with *E. coli *MG1655, coculture with EcN significantly increased release of MCP-1 protein (Fig. 3), indicating an EcN specific up-regulation of this proinflammatory molecule. Additionally, EcN treatment of Caco-2 cells resulted in an increased secretion of IP-10 (interferon gamma-inducible cytokine IP-10; data not shown).

### EcN specific up-regulation of MCP-1 and MIP-2α is not a Caco-2 cell specific phenomenon

**Figure 4:**
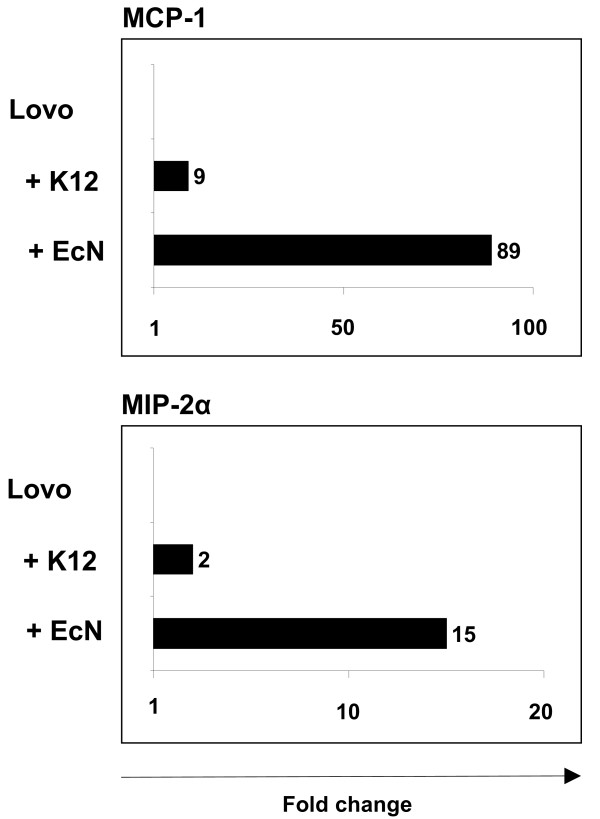
**Gene expression of MCP-1 and MIP-2α in Lovo cells. **Validation of mRNA expression levels of Lovo cells after 6 hours coculture with *E. coli *MG1655 (+ K12) or EcN (+ EcN). Relative mRNA expression levels for MCP-1, MIP-2α and RPS-9 (as internal control) were analyzed in duplicate real-time RT-PCR assays. Relative mRNA amounts were normalized with respect to expression levels of untreated Lovo cells (fold change = 1). The figure is representative of three independent experiments. MCP-1 indicates chemoattractant protein-1 ligand 2; MIP-2α, macrophage inflammatory protein-2 alpha.

To exclude that the EcN specific up-regulation of proinflammatory molecules MCP-1 and MIP-2α was a feature specific for Caco-2 cells, coculture experiments were performed with the human colon adenocarcinoma cell line Lovo. Therefore, confluent Lovo cells were cocultured with EcN or *E. coli *MG1655 for 6 hours, respectively. As already documented for Caco-2 cells, EcN again provoked a strong specific up-regulation of these two proinflammatory molecules. The gene expression level of MCP-1 was 89 fold higher compared to control cells, whereas treatment with *E. coli *MG1655 resulted in a 9 fold increase (Fig. 4). In addition, MIP-2α was 15 fold up-regulated after treatment with EcN in comparison to a 2 fold elevation mediated by *E. coli *MG1655. Results obtained from conventional RT-PCR also revealed an EcN specific induction of MIP-2β (data not shown). Consequently, up-regulation of MCP-1 and MIP-2α after treatment with EcN was not a feature of one cell line but rather a general EcN specific effect on human intestinal epithelial cells.

### Gene expression of MCP-1, MIP-2α and MIP-2β is time-dependent

**Figure 5:**
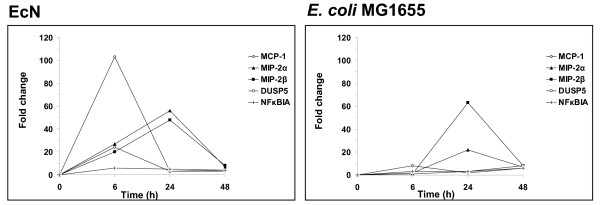
**Time-dependency of the gene expression profile of Caco-2 cells treated with EcN. **Caco-2 cells were cocultured with EcN and *E. coli *MG1655 for 6, 24 and 48 hours. Gene expression at indicated time points was measured by real-time RT-PCR.

Subsequently, we measured time kinetics of gene expression of selected proinflammatory molecules. Extended treatment of Caco-2 cells with EcN or *E. coli *MG1655 for another 24 or 48 hours revealed that EcN-mediated gene expression of MCP-1, DUSP5 and NFκBIA peaked after 6 hours and returned to base levels after 24 hours (Fig. 5). In contrast, expression of MIP-2α and MIP-2β increased further and reached maximum values after 24 hours. However, longer incubation with EcN resulted again in a decreased MIP-2α and MIP-2β gene expression. *E. coli *MG1655 influenced the expression of most of the selected genes just slightly at any time, but revealed a peak for MIP-2β expression after 24 hours, similar to EcN (Fig. 5), suggesting that the observed time course of regulation of this proinflammatory cytokine was not EcN specific.

### EcN induced expression of MIP-2α and MIP-2β is not dependent on viable bacteria

We also aimed to elucidate whether the observed EcN specific expression of selected genes was merely a result of contact between bacterial surfaces and Caco-2 cells and additionally, whether the secretion of active bacterial metabolites was required for the EcN specific induction of the selected genes. Hence, Caco-2 cells were either cocultured with inactivated bacteria or conditioned media (CM) as described in Materials and Methods. Our results indicated that culturing of Caco-2 cells with bacteria CM did not lead to an EcN specific regulation of the selected genes in Caco-2 cells (Tab. 3). Thus, EcN apparently did not secrete active metabolites responsible for specific gene expression. Additionally, for the induction of MCP-1 and NFκBIA gene expression (Fig. 2B) live bacteria were necessary, as no EcN specific gene expression could be measured after exposure of Caco-2 cells to inactivated bacteria (Tab. 3). In contrast, mRNA levels achieved by incubation with formalin killed EcN for MIP-2α, MIP-2β, EMP3, DUSP5 and ELF3 were equivalent to live bacteria. Incubation over 24 h (data not shown) led to a decrease of EcN specific gene expression correlating with the data presented in figure 5. These results suggested that the effect of EcN on the gene expression profile of Caco-2 cells was not only dependent on one single pathway but a combined action of viable bacteria and membrane bound or secreted factors.

### MCP-1 is up-regulated in small intestine after EcN treatment

**Figure 6:**
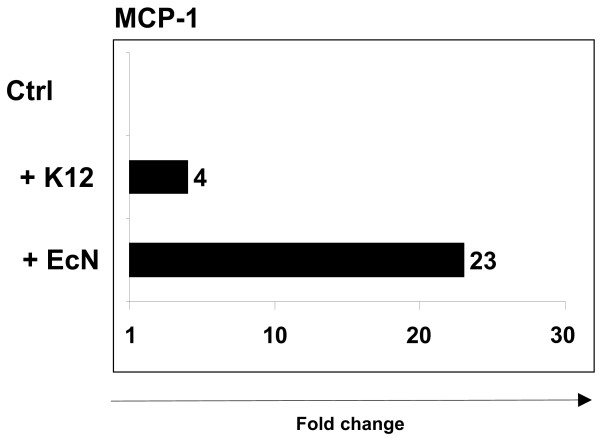
**MCP-1 gene expression in small intestine. **After smooth removal of the mucus layer, 3–5 tissue pieces from small intestine were cocultured with *E. coli *MG1655 (+ K12) or EcN (+ EcN) for 6 hours. Relative mRNA amounts were normalized with respect to expression levels of tissue pieces cultured in IMDM + 10% fetal calf serum without antibiotics (fold change = 1). The figure is representative of three independent experiments.

Our results indicate that the EcN mediated expression of proinflammatory molecules was a short- time effect (Fig. 5). After investigation of the influence of EcN on human cell lines, we next examined the effect of EcN on mouse intestinal epithelial layer. Cima et al. described an efficient method for the differential isolation of intestinal epithelial cells along the villus-crypt axis of the small intestine [23]. Using a modification of this method, pieces of small intestine were cocultured with EcN or *E. coli *MG1655 for 6 hours to analyze the impact of EcN on mouse primary tissue culture. Several reasons prompted us to use small intestinal tissue for these experiments. Since contact between bacteria and intestinal epithelial cells was a required condition for the impact of EcN, a smooth removal of the mucus layer was necessary. This is ensured for small intestine as it has a thinner mucus layer than the colon [26]. Additionally, the murine small intestine is nearly bacteria-free in contrast to a high bacterial density in the colon [26]. Thus, the use of small intestine for primary tissue culture enabled a specific bacteria-cell interaction in contrast to the colon. In order to maintain an intact cell organization and to prevent any stress that might influence the cellular gene expression profile, small tissue pieces were used. Real-time RT PCR revealed a 23 fold increase of MCP-1 expression after treatment with EcN compared to non-treated tissue (Fig. 6). In contrast to EcN, coculture with *E. coli *MG1655 yielded an elevated mRNA level of only 4 fold confirming our results obtained from experiments with confluent human cell lines. Summarized, our data obtained from primary mouse tissue culture confirmed the data of cell culture experiments with two human intestinal epithelial cell lines and corroborated the finding that EcN was able to specifically provoke a proinflammatory host response.

## Discussion

Our results demonstrate that EcN up-regulates gene expression of molecules involved in pro- as well as antiinflammatory processes. A proinflammatory response of intestinal epithelial cells to EcN was mainly demonstrated by the temporary up-regulation of MCP-1 gene expression in confluent human cell lines and in primary mouse tissue culture as well as by an increased secretion of this chemokine. This EcN specific change of MCP-1 gene expression could neither be induced with bacteria conditioned media nor with formalin inactivated organisms suggesting that for EcN mediated MCP-1 gene expression viable bacteria are necessary. MCP-1 is produced by many cells, including epithelial, endothelial and mast cells as well as tumor cells, and shows chemotactic activity for monocytes, basophils, natural killer cells and T lymphocytes during inflammation. Among other things, MCP-1 influences the release of specific enzymes of these target cells [27-30]. MCP-1 is expressed under many pathological conditions, including asthma and inflammatory bowel diseases [31,32]. Infection of human macrophages, which have a central role in innate immune response to bacteria, with non-pathogenic *Lactobacillus rhamnosus *GG, results in an enhanced MCP-1 expression and an induction of T helper cell type 1 (Th1) cell migration [33]. Earlier this year, Lan et al. have shown that exposure of primary murine colonic epithelial cells to the same *Lactobacillus *strain also resulted in an exclusive induction of MCP-1 and MIP-2α expression [34]. Moreover, it has been reported, that lactobacilli are able to activate myeloid dendritic cells that skew CD4^+ ^and CD8^+ ^T cells to Th1 and Tc1 polarization [35]. Earlier studies have shown that MCP-1 expression also shifts the immune response towards release of Th2 cytokines [36] and thus can potentially affect antibacterial defenses. Although it seems unexpected at first sight that the probiotic EcN up-regulates MCP-1 mRNA expression and protein secretion, an important role of this proinflammatory molecule might be established in protecting the host from bacterial infection through induction of a strong T cell immune response, thus providing a reasonable explanation for the EcN induced expression of this chemokine. The neutrophil attracting chemokines MIP-2α and MIP-2β represent two additional genes involved in inflammatory processes and are exhibiting a number of immunoregulatory activities. Both of which showed an increased expression in our coculture experiments after 6 hours, reaching peak levels after 24 hours. Subcutaneous injection of MIP-2 into footpads of C3H/HeJ mice elicited an inflammatory response characterized by neutrophil infiltration, suggesting MIP-2 to be an endogenous mediator playing a role in the host response during inflammatory processes [37]. The MIP-2 expression of lipopolysaccharide (LPS) or IL-1β stimulated intestinal epithelial cells is amplified by butyrate, a metabolite of nonpathogenic, resident bacteria. This is a potential mechanism by which resident bacteria may regulate inflammatory processes in the small intestine [38]. In contrast to MCP-1, EcN mediated expression of MIP-2α and MIP-2β seemed to be independent on the viability of the probiotic in our experiments, as inactivated bacteria induced gene expression as well. Thus, it appears that EcN can induce a potent proinflammatory cytokine response which is in part dependent on the viability of the bacteria. It can be speculated that the local induction of proinflammatory immune responses within the intestinal immune system characterized by up-regulation of MCP-1, MIP-2α and MIP-2β upon contact with probiotic EcN might reflect being part of the host defense process against pathogenic bacteria through the establishment of a protective immunological barrier. However, further *in vitro *and especially *in vivo *experiments are needed to provide evidence that such mechanisms are part of the repertoire of protective responses exhibited by EcN in the intestine.

The transcription factor NF-κB and the corresponding pathway play an important role in proinflammatory signaling [39,40]. Prevention of the NF-κB translocation into the nucleus by the IκB proteins NFκBIA and NFκBIB leads to inhibition of NF-κB, followed by suppression of proinflammatory genes. In our experiments up-regulated gene expression of the transcription inhibitor NFκBIA could be observed. It can be assumed that the observed EcN specific chemokine expression is induced via a NF-κB independent pathway. The JNK/AP-1 signaling cascade is a likely candidate, since transcription and secretion of MCP-1 can also be activated through this pathway triggered by TLR4 signaling [41]. It is a well-known fact that toll-like receptor 4 (TLR4) and 5 (TLR5) recognize LPS [42] and bacterial flagellin [43,44], respectively. Both receptors induce among others the transcription of MCP-1, MIP-2α and MIP-2β [45]. Induction of DSS colitis in TLR4^-/- ^mice led to an altered neutrophil recruitment due to diminished MIP-2 expression by lamina propria macrophages. Thus, TLR4 participates in the intestinal immune response to luminal bacteria and the development of colitis [46]. Since both, EcN and *E. coli *MG1655 possess flagellin and LPS, it can be speculated that an additional component of EcN not yet identified is able to induce MCP-1 gene expression. However, the semirough O6 lipopolysaccharide phenotype of EcN, which is different from O6 LPS of uropathogenic *E. coli *and which is responsible for serum sensitivity of EcN [19], might also play a role in this context.

Even though the proinflammatory activity of EcN, manifested by MCP-1 gene expression and protein secretion, appears surprising at first sight, the genome content of this probiotic strain offers explanations for its proinflammatory properties. Genome comparisons of probiotic EcN and the uropathogenic *E. coli *(UPEC) strain CFT073 as recently published by our laboratory [47] and Grozdanov et al. [17] revealed a high degree of homology between these two strains. The existence of about 130 "common virulence factors" in probiotic EcN, as defined by the PRINTS protein fingerprint database, suggests that these proteins cannot be considered as virulence factors per se, but may also contribute to the general fitness of EcN [47].

## Conclusion

The results of our study revealed that EcN specifically upregulated genes in confluent human intestinal epithelial cell culture. The majorities of these genes were proinflammatory cytokines or belonged to inflammatory pathways. These surrogate *in vitro *markers of probiotic activity of EcN need to be corroborated in other model systems to test their *in vivo *relevance. Additionally, the cell culture model described here will serve as a readout, to evaluate the impact of potential probiotic genes and gene products of EcN, which will consecutively be identified, when our genome sequencing project is finished.

## List of abbreviations used

CFU, colony forming units; CM, conditioned media; DUSP5, dual specificity phosphatase 5; *E. coli *Nissle 1917, EcN; ELF, E74-like factor 3, ets domain transcription factor, epithelial-specific; EMP3, epithelial membrane protein 3; MCP-1, chemoattractant protein-1 ligand 2; MIP-2α, macrophage inflammatory protein-2 alpha; MIP-2β, macrophage inflammatory protein-2 beta; MOI, multiplicity of infection; NFκBIA, nuclear factor of kappa light polypeptide gene enhancer in B cell inhibitor, alpha; Th1, T helper cell type 1; TNFαIP3, tumor necrosis factor, alpha-induced protein 3; TNF, tumor necrosis factor; VEGF, vascular growth factor.

## Competing interests

The author(s) declare that they have no competing interests.

## Authors' contributions

SNU carried out all of the experiments reported except for DNA microarray hybridization, was involved in the interpretation of data, designed figures and tables and drafted the manuscript. AW was involved in design and performance of the study and helped to draft the manuscript. MR carried out electron microscopy. WH was responsible for primer design and participated in real-time RT-PCR analysis. RG performed the statistical analyses and helped with evaluation of microarray data. SC has performed preparatory experiments to this study and was involved in cultivation of all bacterial strains. SS and JB have made substantial contributions to conception of the study and critically revised the manuscript for important intellectual content. FG is primary investigator, who conceived the study, helped to prepare the figures and finished the manuscript. All authors read and approved the final manuscript.

## Pre-publication history

The pre-publication history for this paper can be accessed here:



## Figures and Tables

**Table 1:** Primers used for real-time RT-PCR.

**Product**	**5' Sequence 3'**	**Sense/antisense**	**Product length, bp**	**Tm, °C**
DUSP5	AGC CCC AAG AGC AAC TGT GAT T	Sense	163	54.3
	AGT CCC GAG AAC CTA CCC TGA G	Antisense		
ELF3	GTC AGC AAG CCA GCC CCT ACC AC	Sense	202	62.1
	GGA TCC CCC TTC TTG CAG TCA CGA	Antisense		
EMP3	GCG AGA ATG GCT GGC TGA AG	Sense	204	58.3
	GGA TCT CCT CGG CGT GAA TG	Antisense		
MCP-1	GTC TCT GCC GCC CTT CTG TG	Sense	94	55.9
	AGG TGA CTG GGG CAT TGA TTG	Antisense		
MIP-2α	TTT TAG GTC AAA CCC AAG TTA GTT	Sense	150	49.0
	TTC TTG GAT TCC TCA GCC TCT ATC	Antisense		
MIP-2β	AAG AAG CTT ATC AGC GTA TCA T	Sense	150	50.6
	AAT AAG TAG AAC CCT CGT AAG AAA	Antisense		
NFκBIA	CGC CCA AGC ACC CGG ATA CAG C	Sense	193	58.7
	TTC AGC CCC TTT GCA CTC ATA ACG	Antisense		
RPS-9	CGC AGG CGC AGA CGG TGG AAG C	Sense	92	61.1
	CGA AGG GTC TCC GCG GGG TCA CAT	Antisense		
TNFαIP3	ATT GGC CTC TTT GAT ACA CTT TTG	Sense	201	52.9
	CTC ATC CCT GCT CCT TCC CTA TCT	Antisense		
VEGF	AAG GAG GAG GGC AGA ATC ATC ACG	Sense	192	58.8
	CAC ACT CCA GGC CCT CGT CAT TG	Antisense		

DUSP5 indicates dual specificity phosphatase 5; ELF3, E74-like factor 3 (ets domain transcription factor, epithelial-specific); EMP3, epithelial membrane protein 3; NFκBIA, nuclear factor of kappa light polypeptide gene enhancer in B cell inhibitor, alpha; MCP-1, chemoattractant protein-1 ligand 2; MIP-2α, macrophage inflammatory protein-2 alpha; MIP-2β, macrophage inflammatory protein-2 beta; RPS-9, ribosomal protein S9; TNFαIP3, tumor necrosis factor, alpha-induced protein 3; VEGF, vascular growth factor and Tm, melting temperature.

**Table 2:** Differential gene expression of Caco-2 cells cocultured with EcN.

			**Fold change**^**a**^		
				
**Functional category**	**Gene symbol**	**GB Acc. No.**^**b**^	**Chip 1**	**Chip 2**	**Description and/or putative function**
Immune response	MCP-1 (CCL2)	S69738.1	11.1	3.1	Enhances the inflammatory response, upregulated by IL1α and TNFα
	CD9	NM_001769.1	1.7	3.6	Involved in platelet activation and aggregation; expressed in hematopoetic and epithelial cells
	MIP-2α (CXCL2)	M57731.1	4.4	8.1	Produced by activated monocytes, expressed at inflammation sites, up-regulated by IL1α and TNFα
	MIP-2β (CXCL3)	NM_002090.1	6.5	10.4	Plays a role in inflammation, autocrine effect on endothelial cells
	NK4	NM_004221.1	-1.7	-2.5	May play a role in lymphocyte acitivation, angiogenesis inhibitor, putative therapeutic agent for gastric cancer
					
Signaling	AGT	NM_000029.1	1.7	2.6	Serine protease inhibitor
	CNIH	NM_005776.1	2.6	6.9	EGF-signaling in oocyte
	DUSP5	U16996.1	13.1	3.4	Hydrolase, negative feedback role in IL-2 signaling, deactivation of mitogen- or stress-activated protein kinases
	ID2	NM_002166.1	2.8	4.2	Essential for constitution of the intestinal mucosal barrier, is downregulated by TGFβ
	IFNGR2	NM_005534.1	1.8	2.4	Protein translocator
	NFκBIA	AI078167	6.3	3.3	Downregulation of NF-κB activity, contributed in Crohn's disease
	NFκBIE	NM_004556.1	3.3	2.1	Inhibits NF-κB
	PRDX4	NM_006406.1	1.7	3.6	Regulates the activation of NF-κB by modulation of IκBα phosphorylation
	DDIT (RTP801)	NM_019058.1	20.9	4.0	HIF-1α responsive proapoptotic protein
	TNFαIP3	NM_006290.1	4.3	3.3	Interacts with NAF1 and inhibits TNF-induced NF-κB dependent gene expression
	SFN	X57348	-1.6	-2.2	Exonuclease specific for small oligoribonucleotides, sensitive epithelial marker
					
Transcription and translation	ELF3	U73844.1	3.3	1.8	Epithelial specific ets transcription factor, regulates MIP3α expression which is NF-κB dependent
	ILF3	BC003086.1	-4.3	-2.0	May faciliate ds RNA-regulated gene expression
	TIEG	NM_005655.1	2.5	44.7	Krüppel-like transcription factor, up-regulated by TGFβ
	XBP1	NM_005080.1	2.5	2.7	May act as transcription factor in B-cells
					
Apoptosis	BNIP3	U15174.1	3.0	2.7	Apoptosis inducing protein, binding to BCL2
Differentiation	NDRG1	NM_006096.1	2.4	6.8	Growth inhibitory role, induced app. 20 × during *in vitro *differentiation of Caco-2
Proliferation	EMP3	NM_001425.1	-2.2	-4.4	Probably involved in cell proliferation
	VEGF	H95344	2.1	2.2	Induced by mek1, role of vascular endothelial growth factor in IBD

^a ^Fold change is the factor of mRNA regulation from EcN-treated cells and non-treated cells according to the signal values itself and to the change value from Affymetrix analysis software. Minus fold change values indicate genes with decreased transcription. Results have been performed by two independent hybridization experiments (Chip 1, Chip 2).

^b ^GB Acc. No. indicates GenBank accession number.

**Table 3:** EcN specific expression of selected genes in Caco-2 cells cocultured with inactivated bacteria or bacteria conditioned media for 6 hours.

**Gene**	**Inactivated EcN**	**EcN CM**
DUSP5	**(+)**	no EcN specific gene regulation
ELF3	**(+)**	no EcN specific gene regulation
MCP-1	no EcN specific gene regulation	no EcN specific gene regulation
MIP-2α	**(+++)**	no EcN specific gene regulation
MIP-2β	**(++)**	no EcN specific gene regulation
NFκBIA	no EcN specific gene regulation	no EcN specific gene regulation

Gene expression was analyzed by real-time RT-PCR and differences of fold changes for EcN and *E. coli *MG1655 were compared. EcN specific fold change of Caco-2 cell gene expression (+) between 0.5 and 2, (++) between 2 and 10 and (+++) > 10.
